# Supplementary material for: Modeling glioblastoma heterogeneity as a dynamic network of cell states
Source: Mol Syst Biol. 2021 Sep 16;17(9):e10105. doi: 10.15252/msb.202010105 (PMC8444284; doi:10.15252/msb.202010105)
Supplement: Supplementary file 6 — Source Data for Figure 5 [file MSB-17-e10105-s004.zip › Figure5A_sourcedata/GSEA_3071/nosigdb_stateIV.GseaPreranked.1622795164626/ANASTASSIOU_MULTICANCER_INVASIVENESS_PMID_22208948.html]

Details for gene set ANASTASSIOU\_MULTICANCER\_INVASIVENESS\_PMID\_22208948[GSEA]

|  || Dataset | state53071 |
| Phenotype | NoPhenotypeAvailable |
| Upregulated in class | na\_neg |
| GeneSet | ANASTASSIOU\_MULTICANCER\_INVASIVENESS\_PMID\_22208948 |
| Enrichment Score (ES) | -0.7915355 |
| Normalized Enrichment Score (NES) | -3.3321192 |
| Nominal p-value | 0.0 |
| FDR q-value | 0.0 |
| FWER p-Value | 0.0 |
Table: GSEA Results Summary

  

Fig 1: Enrichment plot: ANASTASSIOU\_MULTICANCER\_INVASIVENESS\_PMID\_22208948      
 Profile of the Running ES Score & Positions of GeneSet Members on the Rank Ordered List

  

| PROBE | GENE SYMBOL | GENE\_TITLE | RANK IN GENE LIST | RANK METRIC SCORE | RUNNING ES | CORE ENRICHMENT || 1 | COL1A2 |  |  | 137 | 1.425 | -0.0022 | No |
| 2 | PRRX1 |  |  | 653 | 0.689 | -0.0886 | No |
| 3 | RAB31 |  |  | 864 | 0.574 | -0.1190 | No |
| 4 | TMEM158 |  |  | 1588 | 0.369 | -0.2504 | No |
| 5 | OLFML2B |  |  | 3411 | -0.392 | -0.5907 | No |
| 6 | RCN3 |  |  | 3635 | -0.434 | -0.6259 | No |
| 7 | LGALS1 |  |  | 3692 | -0.443 | -0.6291 | No |
| 8 | ITGBL1 |  |  | 4095 | -0.539 | -0.6966 | No |
| 9 | ASPN |  |  | 4226 | -0.574 | -0.7117 | No |
| 10 | EDNRA |  |  | 4232 | -0.575 | -0.7030 | No |
| 11 | VCAN |  |  | 4464 | -0.661 | -0.7359 | No |
| 12 | THY1 |  |  | 4587 | -0.729 | -0.7468 | No |
| 13 | LOXL2 |  |  | 4816 | -0.881 | -0.7755 | Yes |
| 14 | NUAK1 |  |  | 4895 | -0.959 | -0.7742 | Yes |
| 15 | TNFAIP6 |  |  | 4987 | -1.072 | -0.7735 | Yes |
| 16 | GLT8D2 |  |  | 5046 | -1.179 | -0.7648 | Yes |
| 17 | NTM |  |  | 5052 | -1.188 | -0.7458 | Yes |
| 18 | MFAP5 |  |  | 5068 | -1.222 | -0.7281 | Yes |
| 19 | COPZ2 |  |  | 5071 | -1.227 | -0.7079 | Yes |
| 20 | COL5A2 |  |  | 5097 | -1.282 | -0.6912 | Yes |
| 21 | SERPINF1 |  |  | 5148 | -1.414 | -0.6769 | Yes |
| 22 | LOX |  |  | 5149 | -1.418 | -0.6531 | Yes |
| 23 | BGN |  |  | 5160 | -1.486 | -0.6301 | Yes |
| 24 | COL11A1 |  |  | 5169 | -1.526 | -0.6060 | Yes |
| 25 | CDH11 |  |  | 5218 | -1.759 | -0.5856 | Yes |
| 26 | DCN |  |  | 5237 | -1.928 | -0.5567 | Yes |
| 27 | SNAI2 |  |  | 5238 | -1.940 | -0.5241 | Yes |
| 28 | PCOLCE |  |  | 5247 | -2.050 | -0.4912 | Yes |
| 29 | PDGFRB |  |  | 5253 | -2.141 | -0.4563 | Yes |
| 30 | COL6A2 |  |  | 5258 | -2.256 | -0.4191 | Yes |
| 31 | FBN1 |  |  | 5263 | -2.334 | -0.3807 | Yes |
| 32 | LUM |  |  | 5271 | -2.487 | -0.3403 | Yes |
| 33 | COL5A1 |  |  | 5276 | -2.605 | -0.2974 | Yes |
| 34 | INHBA |  |  | 5280 | -2.740 | -0.2519 | Yes |
| 35 | FN1 |  |  | 5284 | -3.017 | -0.2019 | Yes |
| 36 | COL3A1 |  |  | 5287 | -3.508 | -0.1433 | Yes |
| 37 | COL6A3 |  |  | 5288 | -3.654 | -0.0820 | Yes |
| 38 | COL1A1 |  |  | 5290 | -4.897 | -0.0000 | Yes |
Table: GSEA details [plain text format]

  

Fig 2: ANASTASSIOU\_MULTICANCER\_INVASIVENESS\_PMID\_22208948: Random ES distribution      
 Gene set null distribution of ES for **ANASTASSIOU\_MULTICANCER\_INVASIVENESS\_PMID\_22208948**
